# Adaptation of emotional state and standing balance parameters following repeated exposure to height-induced postural threat

**DOI:** 10.1038/s41598-019-48722-z

**Published:** 2019-08-28

**Authors:** Martin Zaback, Allan L. Adkin, Mark G. Carpenter

**Affiliations:** 1 School of Kinesiology, University of British Columbia, Vancouver, British Columbia Canada; 2 Department of Kinesiology, Brock University, St. Catharines, Ontario Canada; 3 Djavad Mowafaghian Centre for Brain Health, University of British Columbia, Vancouver, British Columbia Canada; 4 International Collaboration on Repair Discoveries, University of British Columbia, Vancouver, British Columbia Canada

**Keywords:** Motor control, Emotion, Human behaviour

## Abstract

Height-induced postural threat influences standing balance control. However, it is unknown if minimizing individuals’ emotional response to threat moderates this relationship. This study repeatedly exposed individuals to height-induced postural threat to determine if reducing the emotional response to threat influences standing balance control. Sixty-eight young adults completed a series of standing trials at LOW (0.8 m above ground, away from edge) and HIGH (3.2 m above ground, at edge) postural threat conditions. Emotional state was assessed using self-report and electrodermal measures. Standing balance was assessed through analysis of centre of pressure (COP) movement and lower leg electromyographic activity. Individuals’ emotional response to threat was attenuated following repeated threat exposure. However, threat-induced changes in standing balance were largely preserved. When initially threatened, individuals leaned backward and demonstrated smaller amplitude and higher frequency of COP adjustments; these balance outcomes did not change following repeated threat exposure. Only high frequency COP oscillations (>1.8 Hz) and ankle muscle co-contraction showed any adaptation; regression analyses showed that these behavioural adaptations were accounted for by a combination of emotional and cognitive state changes. This suggests that some threat-induced standing balance changes are more closely linked with the emotional response to threat than others, and are therefore amendable to intervention.

## Introduction

Fear of falling is prevalent in older adults^1^ and individuals with movement disorders^2,3^. Cross-sectional studies have shown that older adults with a fear of falling demonstrate differences in standing^4^, reactive^5^, and anticipatory^6^ postural control compared to non-fearful individuals matched for age and level of physical function. These observations, combined with neuroanatomical evidence of direct connections between networks responsible for emotional processing and sensorimotor control of balance^7^, suggest that fear of falling may directly influence balance control, potentially contributing to the increased risk of falling documented amongst these individuals^8–10^.

To examine how fear of falling influences balance control independent of ageing and pathology, research has exposed healthy individuals to different postural threats^11–13^. The most common approach has involved elevating the height of the surface on which individuals stand^11,14–16^. When standing at the edge of an elevated platform, individuals demonstrate a robust emotional response; there are increases in state anxiety and sympathetic arousal (typically estimated from tonic electrodermal activity; EDA) and reductions in balance specific self-efficacy^17–22^. In addition, individuals demonstrate broad changes in attention, directing more attention toward the internal mechanics of their movement, threat-related stimuli, and strategies to regulate their emotional state^23^. These changes in emotional and cognitive state are accompanied by stereotyped changes in standing balance control^24^. Individuals typically lean backwards and demonstrate stiffer control of balance characterized by smaller amplitude and higher frequency postural adjustments and increased co-contraction of lower leg muscles^14,16–22^.

Given the potential links between fear of falling and balance deficits and falls^8–10^, it is important to understand if individuals’ emotional response to a perceived threat can be attenuated and if there are meaningful changes in postural control as a result. One way to explore this is through repeated threat exposure. When repeatedly exposed to a fear-provoking stimulus, individuals typically demonstrate progressive reductions in their emotional response^25^. This has been attributed partially to habituation, a non-associative learning process whereby individuals’ responsiveness to a particular stimulus is reduced following repeated or prolonged exposure^26^. Assuming no real or perceived harm is experienced over the course of repeated threat exposure, associative learning processes also contribute to fear reduction, as individuals’ expectations about the likelihood and severity of feared consequences are gradually disconfirmed^25^. Previous work has shown that repeated exposure to height can substantially reduce the psychological and social consequences associated with a fear of heights^27–30^. However, little attention has focused on how individuals’ postural behaviour is affected following this form of intervention.

Thus, the primary aim of this study was to determine if repeated exposure to a height-induced postural threat influences threat-induced changes in standing balance control. It was hypothesized that after repeated threat exposure, balance confidence would increase and fear of falling, state anxiety, sympathetic arousal, and attention toward one’s movements, threat-related stimuli, and self-regulatory strategies would decrease. These changes in emotional and cognitive state were expected to be accompanied by changes in standing balance control. In particular, after repeated threat exposure, individuals were expected to lean less far away from the platform edge and demonstrate larger amplitude and lower frequency postural adjustments and less ankle muscle co-contraction.

A secondary aim of this study was to explore associations between threat-induced changes in emotional and cognitive state and standing balance control. Previous studies have shown inconsistent correlations between individuals’ emotional response to threat and changes in balance control, with specific balance outcomes (i.e., average frequency of postural adjustments) showing more consistent correlations than others (i.e., amplitude of postural adjustments)^17,19–21^. This suggests that some threat-induced changes in balance control are more closely linked with individuals’ emotional response to threat than others. Alternatively, since the emotional response to threat is multifaceted, with changes in anxiety, arousal, and attention not necessarily varying in lockstep^31^, bivariate correlations may not adequately identify associations between the emotional response to threat and changes in balance control. Thus, this study aimed to determine if a linear combination of emotional and cognitive state changes could account for variance in different balance outcomes when initially threatened, and after having been repeatedly exposed to threat.

## Methods

### Participants

Sixty-eight healthy young adults (mean age ± SD: 22.95 ± 4.06 years; 36 females) participated in this study. Participants were free of musculoskeletal and neurological disorders that could influence balance control. No participants self-reported having an extreme fear of heights. The University of British Columbia Clinical Research Ethics Board approved the experimental procedures, which accorded with the Declaration of Helsinki. All participants provided written informed consent.

### Procedures

**Figure 1:**
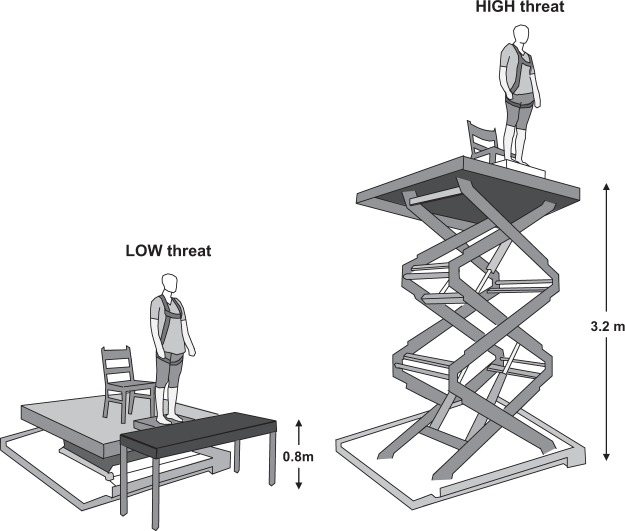
Schematic illustrating the LOW and HIGH postural threat conditions. At the LOW threat condition, participants stood at an elevation of 0.8 m. To minimize anxiety at this condition, an additional support surface (0.6 m × 1.52 m) was positioned in front of, and flush with, the platform edge, creating 60 cm of continuous support surface in front of the participant^16^. At the HIGH threat condition, participants stood at an elevation of 3.2 m and were positioned directly at the edge of the platform. At both threat conditions, participants wore a harness that was secured to the ceiling.

Participants stood barefoot on a force plate (40 cm × 60 cm; AMTI, USA) positioned at the edge of a 2.13 m × 1.52 m hydraulic lift (Penta-lift, Canada) and completed five two-minute trials of quiet standing under two conditions of height-induced postural threat (Fig. 1). Throughout all trials, participants stood with their toes aligned to the anterior edge of the force plate with a stance width equal to their foot length. The borders of the participants’ feet were traced onto the force plate to ensure foot position was consistent across all trials. For the LOW threat condition, the hydraulic lift was positioned at its lowest height (0.8 m above the ground). To minimize anxiety at this condition, an additional support surface (0.6 m × 1.52 m) was positioned in front of, and flush with, the anterior edge of the force plate, creating 60 cm of continuous support surface in front of the participant^16^. For the HIGH threat condition, the hydraulic lift was elevated 3.2 m above the ground and participants stood directly at the platform edge.

Participants were instructed to stand quietly with their arms at their sides and fixate on a visual target 3.87 m in front of them. To maximize adaptation to threat, the five quiet standing trials were completed in a blocked order at each threat condition^30,32^. Two-minutes of seated rest were given between trials, during which time participants completed questionnaires to assess emotional and cognitive state (see *Data collection*). To minimize possible order effects, approximately half of the participants completed the LOW condition first (n = 37), while the others completed the HIGH condition first. Throughout all trials, participants wore a harness secured to the ceiling. The harness did not provide support that could assist in the postural task.

### Data collection

Ground reaction forces and moments were recorded from the force plate and sampled at 100 Hz. Surface electromyography (EMG) was recorded from pairs of Ag/AgCl electrodes placed in bipolar configurations (2 cm inter-electrode distance) over the muscle bellies of the soleus (SOL) and tibialis anterior (TA) of the right leg. A common ground electrode was placed over the lateral malleolus. EMG data were amplified (500×), sampled at 3000 Hz (Telemyo 2400R-G2, Noraxon, USA), bandpass filtered online (10–1000 Hz), and then A-D sampled at 2000 Hz. Electrodermal activity (EDA) was recorded (2502SA, CED, UK) from two Ag/AgCl electrodes placed on the thenar and hypothenar eminences of the non-dominant hand and sampled at 100 Hz. Due to technical issues, EDA was not available for two participants.

Before each trial, participants self-reported their confidence in their ability to maintain balance and avoid a fall during the upcoming balance task. After each trial, participants completed single item self-report questions to assess fear of falling and cognitive and somatic anxiety experienced during the trial. Responses to these questions were provided on visual analog scales ranging from 0 to 100 with graduations marked every 10 units. Higher scores reflected greater balance confidence, fear of falling, and cognitive and somatic anxiety. Scores from the cognitive and somatic anxiety questions were averaged to create a state anxiety score^13^.

Focus of attention was also assessed after each trial using a 5-item questionnaire that asked participants to rate how much they thought about or paid attention to different information throughout each trial^13^. In particular, single questions were used to estimate attention toward (1) movement processes (i.e., conscious control or monitoring of movement; Att. MP); (2) threat-related stimuli (i.e., feelings of anxiety or worry; Att. TRS); (3) self-regulatory strategies (i.e., coping strategies to help remain calm, confident, and/or focused; Att. SRS); (4) task objectives (i.e., focus on specific task instructions; Att. TO); and (5) task-irrelevant information (i.e., thoughts unrelated to the task; Att. TI). Each item was rated on a 9-point Likert scale with higher scores reflecting more attention to each particular loci of attention. The terminology and items used in this questionnaire were developed from open-ended questionnaire and interview data describing the changes in attention associated with height-induced postural threat^23^.

### Data analysis

#### Centre of pressure

Ground reaction forces and moments from the force plate were low-pass filtered offline using a second-order dual-pass Butterworth filter with a cut-off frequency of 10 Hz. From these data, centre of pressure (COP), which reflects the weighted average of pressure applied by the feet onto the support surface^33^, was calculated in the anterior-posterior direction, as postural threat effects are most pronounced in this plane when facing the platform edge^14^. From the COP signal, mean position (MPOS; referenced to the front edge of the force plate) was first calculated to provide an estimate of how far individuals leaned backwards. The MPOS was then subtracted from the COP signal to remove the bias, and a linear detrend was applied to minimize the influence of linear drifts in COP position within each trial that can skew measures of COP amplitude. From the debiased signal, root mean square (RMS) and mean power frequency (MPF) were calculated. RMS reflects the amplitude of COP movement, while MPF reflects the average frequency content contained within the COP power spectrum.

While MPF of COP oscillations typically increases when threatened, it is unclear if this results from a reduction in the power of low frequency COP oscillations, an increase in the power of high frequency COP oscillations, or some combination of the two. Thus, to quantify how COP power changes across different frequencies bands with initial and repeated threat exposure, comparisons of spectra across a bandwidth of relevant COP frequencies (0–5 Hz) were performed^34,35^. For these analyses, COP data were concatenated across participants for the following trials: LOW-1, HIGH-1, and HIGH-5. Power spectral densities of the concatenated data were calculated using a method developed by Halliday and colleagues (1995). Data were first split into equal length, non-overlapping segments (length: 60 s; number of segments: 136). Fast Fourier transformations were applied to each segment, which were then summed and converted to power spectra (frequency resolution: 0.0167 Hz). Log ratios of these power spectra were computed for the following pairs of trials: HIGH-1: LOW-1 (initial threat effect) and HIGH-5: HIGH-1 (adaptation to threat effect). For each comparison, 95% confidence limits were set using an F-distribution based on the number of segments used to generate each power spectra^34^. Frequency bands where COP power significantly differed between conditions were identified. To determine the onset of each band, the frequency bins where data first exceeded a respective confidence limit for at least 3 of 5 consecutive bins were identified. The end of each band was identified as the bin where data returned within the confidence limits for at least 3 of 5 consecutive bins. Mean COP power within specific frequency bands identified were then calculated for each participant (specific frequency bands identified are described in the *Comparison of spectra* section of the *Results*).

#### SOL-TA co-contraction

EMG data for SOL and TA were debiased, full-wave rectified, and normalized to mean rectified EMG of maximal voluntary contractions from respective muscles. These data were linear enveloped using a second-order dual-pass Butterworth filter with a low-pass cut-off of 3 Hz. SOL-TA co-contraction was calculated as the integrated area of overlap between SOL and TA linear envelopes^36,37^.

#### Sympathetic arousal

To provide an estimate of tonic electrodermal activity, the frequency of non-specific electrodermal responses (NS-EDR.freq) were calculated^38^. Electrodermal data were low-passed filtered offline using a fifth-order dual-pass Butterworth filter with a cut-off frequency of 1 Hz. A customized algorithm then identified and counted all EDRs with amplitudes greater than 0.05 ųS^38^. As movement artefacts can be mistakenly identified as EDRs, data were visually inspected and any false positives were removed manually^39^.

### Statistical analyses

A series of 2 (threat: LOW vs HIGH) × 2 (trial: 1 vs 5) repeated measures (RM-) ANOVAs were conducted for self-report (balance efficacy, fear, anxiety, and attention focus), autonomic (NS-EDR.freq), and behavioural measures (MPOS, RMS, MPF, and SOL-TA co-contraction). Significant threat × trial interactions were followed-up with paired-samples t-tests that examined the effect of threat at trials 1 and 5 and the effect of trial for LOW and HIGH threat conditions. Since SOL-TA co-contraction was positively skewed, these data were log-transformed.

To determine if a linear combination of emotional and cognitive state changes could account for changes in behavioural measures when first exposed to the threat, multiple linear regressions were conducted. For these analyses, change scores between the first LOW and HIGH trial (HIGH-1 – LOW-1) were calculated for all emotional, cognitive, and behavioural measures. Change scores for emotional and cognitive state measures were included as independent variables, while change scores for behavioural measures were the dependent variables. A separate set of multiple regressions were conducted to determine if emotional and cognitive state changes could account for changes in behavioural measures over the course of repeated exposure to threat. For these analyses, change scores between trials 1 and 5 of the HIGH threat condition (HIGH-5 – HIGH-1) were calculated for all emotional and cognitive state measures (independent variables), and behavioural measures (dependent variables). Bivariate correlations between emotional and cognitive state measures and behavioural measures were calculated to supplement the multiple regressions. For all statistical tests, alpha was set at 0.05.

## Results

### Emotional and cognitive adaptations

**Figure 2:**
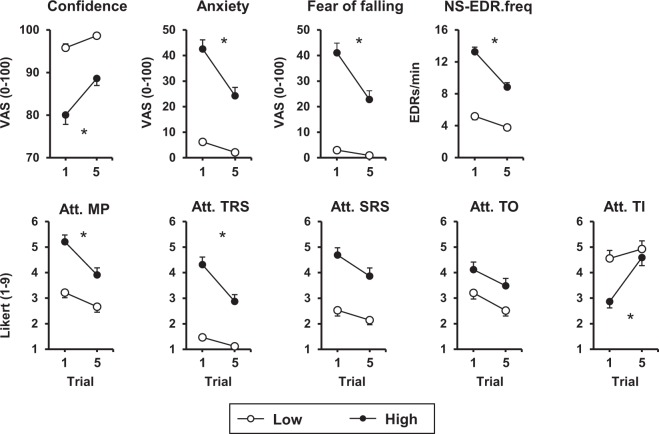
Effect of trial and threat across emotional and cognitive state measures. Group means and standard errors for the first and fifth trials across LOW and HIGH threat conditions are shown. NS-EDR.freq = non-specific electrodermal response frequency; Att. = attention toward; MP = movement processes; TRS = threat-related stimuli; SRS = self-regulatory strategies; TO = task objectives; TI = task irrelevant information. Asterisks indicate significant threat × trial interactions.

Significant threat × trial interactions were observed for measures of balance confidence, anxiety, fear of falling, NS-EDR.freq, and Att. MP, TRS, and TI (all p-values ≤ 0.006; Table 1). Paired t-tests revealed the effect of threat was significant for each of these measures at trial 1, such that individuals were less confident and more anxious, fearful, and physiologically aroused, and directed less attention toward task-irrelevant information and more attention toward movement processes and threat-related stimuli when standing at the HIGH compared to LOW threat. By trial 5, the effect of threat was reduced, but remained significant for all measures except for Att. TI, which returned to LOW threat values. In all cases, the effect of threat was attenuated due to greater changes observed from trial 1 to 5 at the HIGH compared to LOW threat (Fig. 2).

**Table 1:** Summary of statistical test results for 2 × 2 repeated-measures ANOVAs for emotional and cognitive state and behavioural measures.

	Threat × Trial interaction			Threat			Trial		
	F	p	η^2^	F	p	η^2^	F	p	η^2^
**Emotional and cognitive outcomes**									
Balance confidence	**12**.**003**	**0**.**001**	**0**.**152**	**69**.**667**	<**0**.**001**	**0**.**510**	**24**.**290**	<**0**.**001**	**0**.**266**
Anxiety	**37**.**158**	<**0**.**001**	**0**.**357**	**96**.**322**	<**0**.**001**	**0**.**590**	**83**.**855**	<**0**.**001**	**0**.**556**
Fear of falling	**37**.**707**	<**0**.**001**	**0**.**360**	**88**.**014**	<**0**.**001**	**0**.**568**	**58**.**716**	<**0**.**001**	**0**.**467**
NS-EDR.freq	**34**.**637**	<**0**.**001**	**0**.**348**	**200**.**571**	<**0**.**001**	**0**.**755**	**144**.**814**	<**0**.**001**	**0**.**690**
Att. MP	**8**.**017**	**0**.**006**	**0**.**107**	**49**.**253**	<**0**.**001**	**0**.**424**	**35**.**348**	<**0**.**001**	**0**.**345**
Att. TRS	**26**.**517**	<**0**.**001**	**0**.**284**	**86**.**628**	<**0**.**001**	**0**.**564**	**57**.**938**	<**0**.**001**	**0**.**464**
Att. SRS	2.379	0.128	0.034	**62**.**825**	<**0**.**001**	**0**.**484**	**13**.**944**	<**0**.**001**	**0**.**172**
Att. TO	0.034	0.885	0.001	**23**.**985**	<**0**.**001**	**0**.**264**	**15**.**996**	<**0**.**001**	**0**.**193**
Att. TI	**21**.**858**	<**0**.**001**	**0**.**246**	**26**.**136**	<**0**.**001**	**0**.**281**	**37**.**242**	<**0**.**001**	**0**.**357**
**Behavioural outcomes**									
MPOS	0.028	0.867	<0.001	**112**.**477**	<**0**.**001**	**0**.**627**	0.262	0.610	0.004
RMS	1.689	0.198	0.025	**7**.**433**	**0**.**008**	**0**.**100**	0.567	0.454	0.008
MPF	0.221	0.640	0.003	**64**.**226**	<**0**.**001**	**0**.**489**	0.321	0.573	0.005
SOL-TA CC	**23**.**328**	<**0**.**001**	**0**.**258**	**77**.**110**	<**0**.**001**	**0**.**535**	**17**.**716**	<**0**.**001**	**0**.**209**

*Note*. NS-EDR.freq = non-specific electrodermal response frequency; Att. = attention toward; MP = movement processes; TRS = threat-related stimuli; SRS = self-regulatory strategies; TO = task objectives; TI = task-irrelevant information; MPOS = mean position of centre of pressure; RMS = root mean square of centre of pressure; MPF = mean power frequency of centre of pressure; SOL-TA CC = soleus-tibialis anterior co-contraction.

Significant effects are bolded.

Of the remaining emotional and cognitive state measures, significant main effects of threat and trial were observed for Att. SRS and TO. In both cases, individuals directed more attention toward this information at the HIGH compared LOW threat condition (p-values < 0.001) and directed less attention toward this information from trial 1 to 5 independent of threat condition (p-values < 0.001).

### Standing balance adaptations

**Figure 3:**
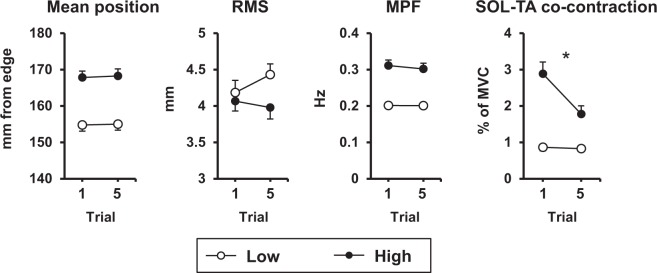
Effect of trial and threat across standing balance measures. Group means and standard errors of standing balance measures for the first and fifth trials across LOW and HIGH threat conditions are shown. RMS = root mean square of centre of pressure; MPF = mean power frequency of centre of pressure; SOL = soleus; TA = tibialis anterior. Asterisks indicate significant threat × trial interactions.

A significant threat × trial interaction was only observed for SOL-TA co-contraction (p < 0.001; Table 1). Paired t-tests revealed the effect of threat was significant for SOL-TA co-contraction at trial 1, such that individuals co-contracted more at the HIGH compared to LOW threat. The effect of threat remained significant by trial 5, but was reduced. Paired t-tests revealed this was due to significant decreases in co-contraction from trial 1 to 5 across only the HIGH threat condition (Fig. 3).

For all other behavioural measures, significant main effects of threat were observed. When standing at the HIGH compared to LOW threat condition, MPOS shifted away from the platform edge, RMS decreased, and MPF increased (p-values ≤ 0.008). For each of these measures, no interactions or main effects of trial were observed, demonstrating that these measures were affected by threat, but did not change over the course of repeated threat exposure.

#### Comparison of spectra

**Figure 4:**
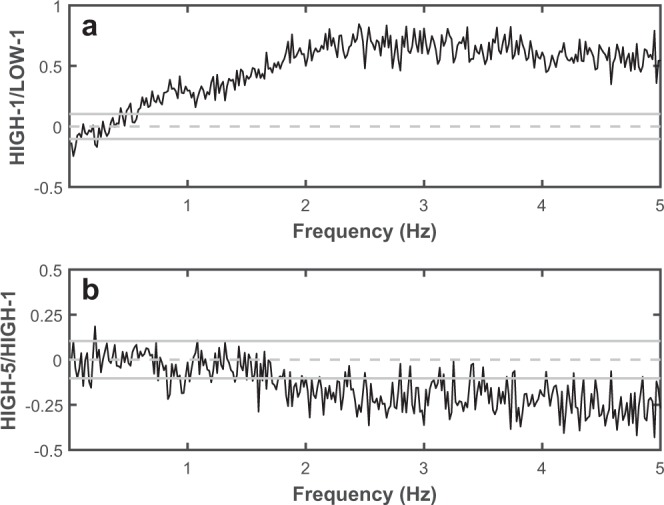
Centre of pressure comparison of spectra analyses illustrating the effects of initial and repeated threat exposure. Data shown reflect the log ratio of COP power spectral density between the first HIGH and LOW trial (**a**) and fifth and first HIGH trial (**b**) across frequencies (resolution 0.0167 Hz). Horizontal grey lines reflect 95% confidence limits.

With initial threat exposure (HIGH-1: LOW-1), COP power was reduced at frequencies lower than 0.05 Hz and was increased at frequencies between 0.48–0.52 Hz and above 0.59 Hz (Fig. 4a). With repeated threat exposure (HIGH-5: HIGH-1), COP power was reduced at frequencies 0.92–0.98 Hz, 1.72–1.77 Hz, and above 1.833 Hz (Fig. 4b).

Based on these results, mean COP power was calculated within the following frequency bands: 0–0.05 Hz (low frequency), 0.59–1.82 Hz (medium frequency), and 1.83–5 Hz (high frequency). The low frequency band was selected as this was the only region where COP power was reduced with threat and this region, while small, contains the most power in the COP power spectrum. The medium frequency band was selected as this was a region where COP power increased with threat, but showed negligible adaptation with repeated threat exposure. The high frequency band was selected as this was a region where COP power increased with initial threat exposure and showed significant adaptation with repeated exposure. Change scores (HIGH-1 – LOW-1 and HIGH-5 – HIGH-1) were calculated for these measures of mean COP power and included as dependent variables in the multiple linear regressions.

### Associations between changes in emotional and cognitive state and standing balance parameters

#### Initial threat exposure

Anxiety and fear of falling had variance inflation factors (VIFs) greater than 2.5, indicating substantial multicollinearity with the other independent variables entered into these multiple regressions. Therefore, these variables were not included as independent variables in these multiple regressions. Thus, each multiple regression included 7 independent variables (balance confidence, NS-EDR.freq, and Att. MP, TRS, SRS, TO, and TI). As shown in Table 2, significant multiple regressions were observed for MPF (R^2^ = 0.465, *p < *0.001), high frequency COP power (R^2^ = 0.337, *p = *0.001), and SOL-TA co-contraction (R^2^ = 0.354, *p < *0.001), showing that a linear combination of emotional and cognitive state changes could account for variance in these behavioural measures when initially threatened. The only significant individual predictor for changes in MPF and high frequency COP power was Att. TRS (MPF: β = 0.542, *p* < 0.001; high frequency COP power: β = 0.420, *p* = 0.007). No significant individual predictors were observed for SOL-TA co-contraction.

**Table 2:** Multiple correlations (R^2^) and standardized beta weights for regressions between changes in emotional and cognitive state measures and behavioural measures.

	MPOS	RMS	MPF	LF power	MF power	HF power	SOL-TA CC
***Initial threat exposure (standardized beta weights)***							
Balance confidence	0.070	0.154	−0.170	0.146	−0.015	−0.102	−0.228
NS-EDR.freq	0.070	0.045	0.031	0.038	0.195	0.100	0.210
Att. MP	0.074	0.253	−0.174	0.253	0.019	0.097	0.153
Att. TRS	0.306	−0.206	0.542***	−0.223	0.199	0.420**	0.138
Att. SRS	0.035	0.030	0.134	0.042	0.108	0.013	0.119
Att. TO	0.147	−0.197	0.105	−0.171	0.015	0.031	−0.071
Att. TI	0.238	−0.167	0.039	−0.084	−0.042	−0.011	−0.191
***Initial threat exposure (multiple correlations (R*** ^***2***^ ***))***							
***R*** ^***2***^	*0.196*	*0.127*	0.456***	*0.106*	*0.184*	0.337**	0.354***
***p-value***	*0.067*	*0.313*	***<0.001***	*0.455*	*0.091*	***0.001***	***<0.001***
***Repeated threat exposure (standardized beta weights)***							
Balance confidence	−0.315*	0.047	−0.198	0.083	−0.011	−0.081	−0.209
Anxiety	−0.437*	0.045	0.008	−0.001	−0.073	0.038	−0.011
NS-EDR.freq	0.171	0.038	−0.049	0.079	0.149	−0.014	0.229
Att. MP	0.241	0.406*	−0.043	0.330*	0.356*	0.376*	0.089
Att. TRS	0.290	−0.245	0.345	−0.162	−0.085	0.311	0.120
Att. SRS	0.051	−0.150	0.142	−0.162	0.139	−0.099	0.169
Att. TO	0.137	−0.065	−0.043	−0.024	−0.047	−0.034	−0.079
Att. TI	0.270*	−0.130	0.237	−0.107	0.204	0.115	−0.198
***Repeated threat exposure (multiple correlations (R*** ^***2***^ ***))***							
***R*** ^***2***^	0.261*	*0.157*	*0.191*	*0.113*	*0.196*	0.268*	0.323**
***p-value***	***0.020***	*0.248*	*0.123*	*0.515*	*0.109*	***0.017***	***0.003***

*Note*. Independent and dependent variables reflect change scores (Initial threat exposure = HIGH1-LOW1; Repeated threat exposure = HIGH5-HIGH1).

NS-EDR.freq = non-specific electrodermal response frequency; Att. = attention toward; MP = movement processes; TRS = threat-related stimuli; SRS = self-regulatory strategies; TO = task objectives; TI = task-irrelevant information; MPOS = mean position of centre of pressure; RMS = root mean square of centre of pressure; MPF = mean power frequency of centre of pressure; LF power = low frequency centre of pressure power; MF power = medium frequency centre of pressure power; HF power = high frequency centre of pressure power; SOL-TA CC = soleus-tibialis anterior co-contraction.

*p < 0.05; **p < 0.01; ***p < *0.001*; significant β’s and R^2^’s are bolded

Results from the bivariate correlations were generally consistent with the multiple linear regression analyses, with changes in MPF, high frequency COP power, and SOL-TA co-contraction showing the strongest and most consistent correlations with a number of emotional and cognitive state changes (Table 3).

**Table 3:** Bivariate correlations between emotional and cognitive and behavioural outcomes when initially threatened and after repeated threat exposure.

	MPOS	RMS	MPF	LF power	MF power	HF power	SOL-TA CC
***Initial threat exposure***							
Confidence	−0.085	0.204	**−0**.374**	0.200	−0.111	**−**0.278*	−0.339**
Anxiety	0.291*	−0.144	0.518***	−0.140	0.327**	0.418**	0.623***
Fear	0.256*	−0.094	0.571***	−0.094	0.388**	0.496**	0.555***
NS-EDR.freq	0.236	−0.101	0.386**	−0.101	0.339**	0.322**	0.378**
Att. MP	0.213	0.140	0.170	0.129	0.174	0.313**	0.317**
Att. TRS	0.382**	−0.100	0.638***	−0.120	0.386**	0.572***	0.488***
Att. SRS	0.311**	0.022	0.444**	0.009	0.379**	0.397**	0.433***
Att. TO	0.078	−0.103	0.090	−0.097	0.037	0.033	0.024
Att. TI	0.097	−0.172	−0.026	−0.098	−0.116	−0.085	−0.198
***Repeated threat exposure***							
Confidence	−0.239*	0.038	−0.153	0.067	0.031	−0.037	−0.162
Anxiety	0.007	−0.009	0.190	−0.013	0.148	0.260*	0.299*
Fear	0.040	−0.020	0.269*	−0.010	0.225	0.323**	0.361**
NS-EDR.freq	0.189	−0.009	0.119	0.020	0.236	0.132	0.374**
Att. MP	0.220	0.254*	0.007	0.222	0.220	0.322**	0.209
Att. TRS	0.131	−0.083	0.242*	−0.048	0.065	0.301*	0.305*
Att. SRS	0.114	−0.014	0.229	−0.025	0.346**	0.273*	0.379**
Att. TO	0.144	0.046	0.027	0.050	0.108	0.152	0.063
Att. TI	0.139	−0.159	0.101	−0.138	0.062	−0.120	−0.282*

*Note*. Variables reflect change scores (Initial threat exposure = HIGH1-LOW1; Repeated threat exposure = HIGH5-HIGH1).

NS-EDR.freq = non-specific electrodermal response frequency; Att. = attention toward; MP = movement processes; TRS = threat-related stimuli; SRS = self-regulatory strategies; TO = task objectives; TI = task-irrelevant information; MPOS = mean position of centre of pressure; RMS = root mean square of centre of pressure; MPF = mean power frequency of centre of pressure; LF power = low frequency centre of pressure power; MF power = medium frequency centre of pressure power; HF power  = high frequency centre of pressure power; SOL-TA CC = soleus-tibialis anterior co-contraction.

*p < *0*.05; **p < *0*.01; ***p < *0*.*001*.

#### Repeated exposure to threat

Fear of falling had a VIF greater than 2.5; therefore, this variable was not entered as an independent variable in these multiple regressions. Thus, each multiple regression included 8 independent variables (balance confidence, anxiety, NS-EDR.freq, and Att. MP, TRS, SRS, TO, and TI). Significant multiple regressions were observed for MPOS (R^2^ = 0.261, *p* = 0.020), high frequency COP power (R^2^ = 0.268, *p* = 0.017), and SOL-TA co-contraction (R^2^ = 0.323, *p* = 0.003), showing that a linear combination of emotional and cognitive state changes could account for variance in these behavioural measures after repeated exposure to threat. Significant individual predictors of changes in MPOS were balance confidence (β = −0.315, *p* = 0.016), anxiety (β = −0.437, *p* = 0.018), and Att. TI (β = 0.270, *p* = 0.043). The only significant individual predictor for high frequency COP power was Att. MP (β = 0.376, *p* = 0.013). No significant individual predictors were observed for SOL-TA co-contraction (Table 2).

With the exception of the model for MPOS, results from the bivariate correlations were generally consistent with the multiple linear regression analyses, with changes in high frequency COP power and SOL-TA co-contraction showing the strongest and most consistent correlations with changes in emotional and cognitive state following repeated threat exposure (Table 3).

## Discussion

The primary aim of this study was to understand how standing balance control adapts following repeated exposure to height-induced postural threat. Consistent with previous research, this study showed that height-induced postural threat induces a robust emotional response that is accompanied by changes in standing balance control; in particular, individuals leaned away from the edge of the platform and showed smaller amplitude and higher frequency COP adjustments^24^. This study was also the first to demonstrate that changes in COP MPF with threat are due to decreases in lower frequency COP components (≤0.05 Hz) and increases in higher frequency COP components (>0.5 Hz). As hypothesized, threat-induced changes in emotional and cognitive state were significantly attenuated following repeated threat exposure. In particular, individuals demonstrated increases in balance confidence and attention toward task-irrelevant information, and reductions in anxiety, fear of falling, sympathetic arousal, and attention toward movement processes and threat-related stimuli. However, with the exception of attention toward task-irrelevant information, these emotional and cognitive outcomes remained elevated above LOW threat values, showing that complete adaptation was not achieved. Contrary to what was hypothesized, few changes in standing behaviour were observed after repeated threat exposure, with changes in high frequency COP power (>1.83 Hz) and SOL-TA co-contraction representing the only behavioural outcomes to show any adaptation across multiple exposures to the HIGH threat condition. Thus, despite significant adaptation of the emotional response to threat, individuals’ behavioural response to threat was largely preserved.

A secondary aim of this study was to further explore associations between threat-induced changes in standing balance and emotional and cognitive state. Changes in several standing balance outcomes were related to a combination of emotional and cognitive state changes when initially threatened as well as after having been repeatedly exposed to threat; this included high frequency COP power (1.83–5 Hz) and SOL-TA co-contraction. Some standing balance outcomes were unrelated to a combination of emotional and cognitive state changes (i.e., RMS and low frequency COP power) or only showed significant relationships with either initial (i.e., MPF) or repeated (i.e., MPOS) threat exposure. These findings are somewhat consistent with previous work. Of the COP outcomes typically assessed in postural threat studies, changes in MPF have been most consistently correlated with emotional and cognitive state changes^17,19–21^. The present study replicated this observation and provides evidence to suggest this is due to a strong link between changes in higher frequency COP components and individuals’ emotional response to threat. By contrast, threat-induced changes in COP amplitude, typically assessed by calculating RMS of COP, are seldom associated with emotional and cognitive state changes^17,20,21^. Even after accounting for the combined influence of multiple emotional and cognitive state changes, threat-induced changes in RMS and lower frequency COP components could not be accounted for. Collectively, these results suggest that some threat-induced changes in standing balance are more closely linked with individuals’ emotional response to threat than others.

### Why were behavioural adaptations limited?

There are several possible explanations for why individuals’ behavioural response to threat was largely preserved following repeated threat exposure. First, it is possible that a non-linear relationship exists between some threat-induced changes in behaviour and individuals’ emotional response to threat, such that considerable or near complete adaptation is needed before some behavioural outcomes show any sign of adaptation. The results from the regression-based analyses can support this speculation, as several behavioural outcomes that did not show adaptation at a group level were significantly related to either a combination of emotional and cognitive outcomes (i.e., MPOS and MPF) or showed several moderate correlations with emotional and cognitive outcomes (i.e., medium frequency COP power). These significant relationships may have been the result of behavioural adaptations starting to manifest in only those individuals who showed the greatest emotional and cognitive adaptation. If this is the case, broader behavioural adaptations may have been observed with a longer exposure period and greater emotional and cognitive adaptation at a group level.

It is also possible that some threat-induced changes in behaviour are primarily context-dependent, such that they are adopted irrespective of individuals’ emotional response to threat. Previous work has shown that the behavioural response to postural threat differs depending on the type of threat^13,40^. In particular, when standing with the threat of an unpredictable forward or backward perturbation, healthy young adults show higher frequency postural adjustments, consistent with what is seen with a height-induced threat, but tend to lean forward and show larger amplitude postural adjustments^13,40^. This difference in postural strategy is observed despite individuals reporting similar emotional and cognitive state changes in both threat scenarios. If some components of the behavioural response to threat are primarily related to the context of the threat, they may be less amendable following repeated threat exposure.

Lastly, some threat-induced changes in behaviour may be associated with state-related changes not measured in the present study. For example, it has been shown that there are broad, multi-sensory changes when individuals are exposed to height-induced threat, whereby balance relevant proprioceptive^41–44^ and vestibular^45–47^ reflexes are facilitated. It is unclear if these changes in sensory processing scale linearly with individuals’ emotional response to threat. In addition, cognitive factors known to influence vestibular reflex gain, such as vigilance or tonic alertness^48,49^, were not accounted for in the present study and may not vary linearly with changes in emotional state as measured in this study. Since threat-induced changes in sensory processing may increase reflex feedback gain and allow for more accurate monitoring of postural state, maintenance of these changes following repeated exposure could explain why some threat-induced changes in behaviour do not show adaptation.

### Functional implications

The threat-induced changes in standing balance that appear most susceptible to adaptation following repeated threat exposure are the highest frequency components of COP movement and ankle muscle co-contraction. These behavioural outcomes are highly correlated to each other (Table 4), with co-contraction likely driving higher frequency COP oscillations^50,51^. These particular components of the behavioural response to threat may be maladaptive for several reasons. First, while slower fluctuations in the COP position have been argued to serve an exploratory role, providing the nervous system with an increased inflow of balance relevant sensory information^52^, very high frequency COP oscillations may interfere with or mask relevant somatosensory input^53,54^. It has been argued that this is why individuals with orthostatic tremor, a disorder characterized by a 13–18 Hz tremor of the lower limbs and trunk during stance, have difficulty processing proprioceptive and cutaneous inputs^55^. High frequency COP oscillations (>2 Hz) also contribute negligibly to the control of the centre of mass during stance due to the large moment of inertia of the centre of mass^56^. Lastly, increased ankle muscle co-contraction may impair muscle spindle coding of ankle joint rotations^57^ and interfere with dynamic balance control^58^. By contrast, other threat-induced changes in standing balance that may serve a more protective role, such as the reduced amplitude of COP movement and lean away from the platform edge, appear less prone to adaptation. The tendency for maladaptive behaviours to be minimized and more protective behaviours to be preserved may be beneficial for interventions designed to reduce fear of falling. For instance, reducing an individual’s fear of falling in a particular scenario may not make them more prone to employing risky postural strategies, but it may reduce their tendency to employ strategies that interfere with sensorimotor processes underlying the control of balance.

**Table 4:** Bivariate correlations between ankle muscle co-contraction and COP outcomes.

	MPOS	RMS	MPF	LF power	MF power	HF power
Initial threat exposure	0.337**	0.064	0.453***	0.026	0.511***	0.531***
Repeated threat exposure	0.334**	0.014	0.346**	−0.007	0.381**	0.459***

*Note*. Variables reflect change scores (Initial threat exposure = HIGH1-LOW1; Repeated threat exposure = HIGH5-HIGH1).

MPOS = mean position of centre of pressure; RMS = root mean square of centre of pressure; MPF = mean power frequency of centre of pressure; LF power = low frequency centre of pressure power; MF power = medium frequency centre of pressure power; HF power = high frequency centre of pressure power.

*p < 0.05; **p < 0.01;***p < 0.*001*.

### Limitations and future directions

The exposure period used in this study was relatively brief and only resulted in a fairly modest reduction of the emotional response to threat at a group level (approximately 50% reduction of the initial effect of threat for most emotional and cognitive outcomes). Future work should use a longer exposure period, either within a single testing session or across multiple sessions, to determine if some components of the behavioural response to threat are still preserved following near complete adaptation of the emotional response.

While this study provides evidence that attenuating the emotional response to a perceived threat is accompanied by specific changes in standing balance control, these results are only generalizable to healthy young adults exposed to a height-induced threat. It is unclear if older adults or patient populations with a fear of falling would show a similar pattern of adaptation following repeated exposure to different scenarios they perceive as threatening to balance. This is an important avenue of future research in order to establish the clinical efficacy of repeated threat exposure as an intervention for individuals who regularly experience a fear of falling.

Lastly, while the regression coefficients (beta weights) for individual predictors entered into the multiple regression models are reported, interpretation of these results has been restrained. While independent variables were screened for extreme collinearity, there was still considerable shared variance amongst them. Mild collinearity can lead to substantial inaccuracies in estimated regression coefficients in modest predictive models (R^2^ > 0.5) with small samples (n < 100)^59^.To accurately parse the unique influence of individual emotional and cognitive factors to specific changes in balance control using a multiple regression approach, a much larger sample is needed^59,60^. Alternatively, future studies may seek to independently manipulate specific emotional and cognitive factors associated with threat to understand which are most important in shaping behaviour.

## Conclusions

Individuals demonstrated modest reductions of their emotional response to threat following a relatively short period of blocked repeated threat exposure. However, threat-induced changes in standing balance control were largely preserved, with higher frequency COP oscillations and ankle muscle co-contraction representing the only behavioural outcomes to show any adaptation. Regression-based analyses demonstrated that these components of the behavioural response to threat are most closely linked with individuals’ emotional response to threat. This suggests that not all threat-induced changes in standing balance control are related, or at least linearly related, to changes in emotional state, emphasizing the need to further explore the mechanisms underlying the relationship between postural threat and standing balance control. Results from this study are promising for the design of interventions for individuals who live with fear of falling (e.g., frail elderly and/or individuals with balance deficits) or work in environments that may induce fear of falling (e.g., construction workers), since attenuating individuals’ emotional response to threat appears to have the greatest effect on the most maladaptive balance control changes, while preserving those that may be more protective in nature.

## Supplementary information

Dataset 1

## Data Availability

All data generated or analyzed during this study are included in the published article (and its Supplementary Data Files).
